# Transient response of the Northwestern Iberian upwelling regime

**DOI:** 10.1371/journal.pone.0197627

**Published:** 2018-05-17

**Authors:** Nuno Gonçalo Ferreira Cordeiro, Jesus Dubert, Rita Nolasco, Eric Desmond Barton

**Affiliations:** 1 Departamento de Física and CESAM, Universidade de Aveiro, Aveiro, Portugal; 2 Instituto de Investigaciones Marinas (CSIC), Vigo, Spain; University of Vigo, SPAIN

## Abstract

The hydrography and dynamics of NW Iberian margin were explored for July 2009, based on a set of in situ and remote sensing observations. Zonal sections of standard CTD casts, towed CTD (SeaSoar), Acoustic Doppler Current Profilers (ADCP) and Lagrangian surveys were made to characterize cycles of upwelling and relaxation in this region. Two periods of northerly winds, bounded by relaxation periods, were responsible for the formation of an upwelling front extending to the shelf edge. An equatorward flow was quickly set up on the shelf responding to the northerly wind pulses. South of Cape Silleiro, the development and subsequent relaxation of an upwelling event was intensively surveyed in the shelf, following a Lagrangian drifter transported by the upwelling jet. This region is part of an upwelling center extending from Cape Silleiro to Porto, where the surface temperature was colder than the neighboring regions, under upwelling favorable winds. As these winds relaxed, persistent poleward flow developed, originating south of the upwelling center and consisting in an inner-shelf tongue of warm waters. During an event of strong southerly wind, the poleward flow was observed to extend to the whole continental shelf. Although the cruise was executed during summertime, the presence of river-plumes was observed over the shelf. The interaction of the plumes with the circulation on the shelf was also described in terms of coastal convergence and offshore advection. The sampling of the offshore and slope regions showed the presence of the Iberian poleward current offshore and a persistent equatorward flow over the upper slope.

## Introduction

The upwelling favorable season in the Western Iberian Margin (WIM) occurs between June and September [1, 2], when the northward shift of the Azores high pressure system produces northerly winds in the region. In spite of the predominance of upwelling during summer, downwelling interludes are frequently observed during wind relaxations associated with the rapid passage of low pressure systems [3], which occur in cycles of 14 days or less [4]. A spring transition to predominance of upwelling occurs around May-June through the establishment of northerly winds and a seasonal thermocline. Flow on the shelf and slope changes from being predominantly poleward in winter to mainly equatorward in the upwelling season [5].

As with other Eastern Boundary Systems, the large scale circulation is dominated by an offshore slow equatorward flow, here the Portugal Current, which forms part of the subtropical gyre recirculation. This flow co-exists with the counter flow of the Iberian Poleward Current (IPC) over the continental slope driven mainly by a meridional density gradient [6]. The IPC is thought to be present all year round, although it weakens and spreads offshore in spring and is confined to the subsurface during the summer, below the upwelling jet [7]. During the early upwelling season, the offshore IPC has been observed to coexist with shelf and slope equatorward flows [3].

**Fig 1:**
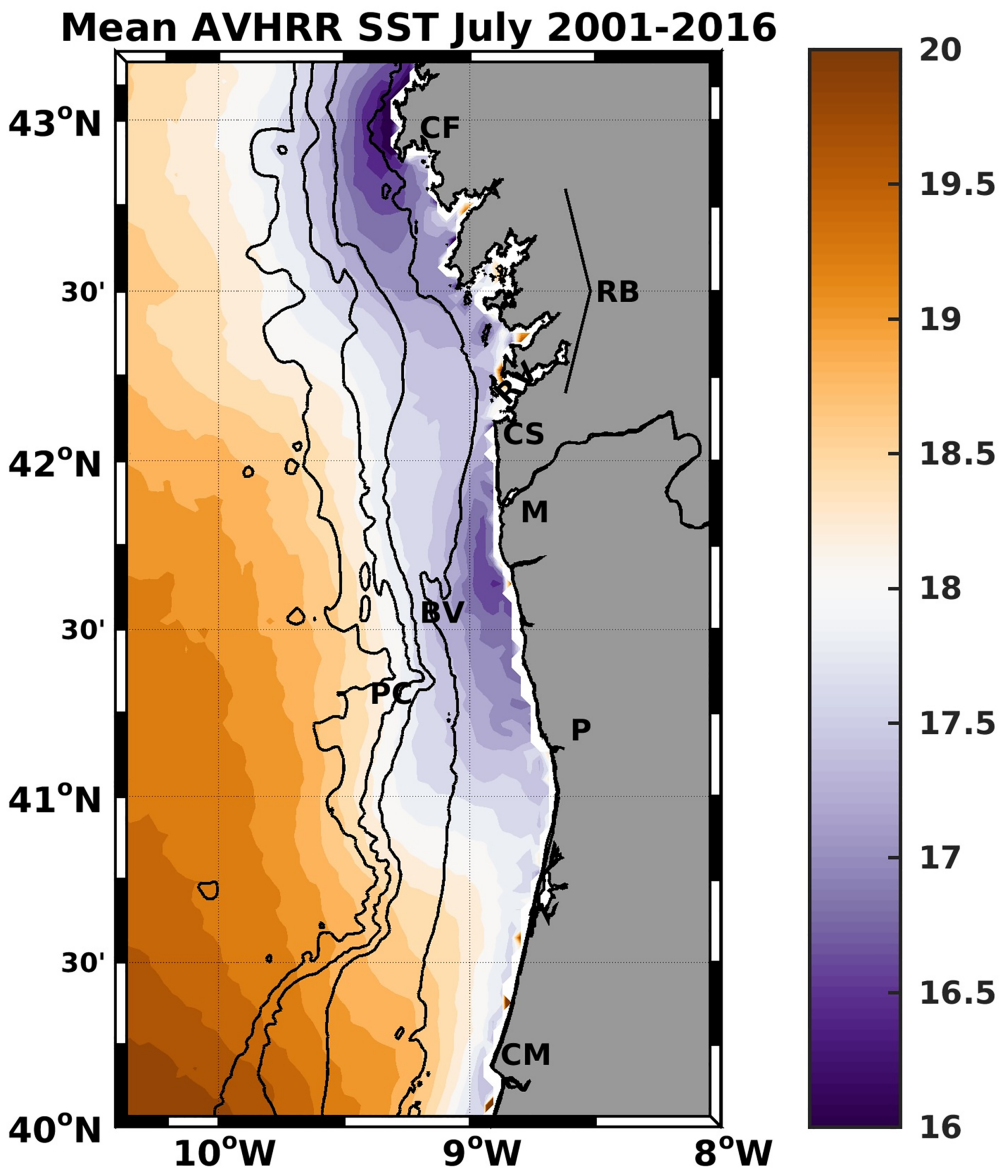
Map of the 2001-2016 July average of SST from AVHRR satellite data. Initials represent the location of coastal features: Cape Finisterre (CF), Rias Baixas (RB), Cape Silleiro (CS), river Minho mouth (M), Porto (P), Cape Mondego (CM), and bathymetric features: Beiral de Viana (BV) and Porto Canyon (PC). The 100-m, 200-m, 1000-m and 2000-m bathymetric depths are represented in dark lines.

The Northwestern Iberian Margin (NWIM) stretches from Cape Mondego to Cape Finisterre (40°N to 43°N in Fig 1). North of Cape Silleiro (42°06’N), the coastline is made up of a series of embayments, the Rías Baixas; to the south, it is relatively smooth and oriented approximately NNW as far as Porto (41°06’N). The shelf deepens gently to the shelf edge at the 200-m isobath, and is bounded by a steep slope that plunges to 2000-4000 m. To the south of Porto Canyon (41°20’N) the shelf is around 50-60 km wide, while to the north it narrows. South of Beiral de Viana (41°40’N) the inner shelf (inshore of 100-m isobath) is wider. The neighborhood of Cape Finisterre and the region between Cape Silleiro and Porto (Fig 1), are known to present centers of intensified upwelling [8], in which coastal upwelled waters are colder than elsewhere. Causes for the existence of intensified local upwelling include the spatial variability of the wind [9] and the shelf width [10]. The upwelling center near Cape Finisterre could also be related with the 90°change in orientation of the coast and the related wind stress curl [11].

In upwelling regions, the continental shelf circulation is typically set up on a scale of hours [12] to days [13] in response to the atmospheric forcing. When northerly winds blow along a meridional coast, surface coastal waters are transported offshore through an Ekman layer, and are replaced by rising of subsurface cold waters. Equatorward flow develops in geostrophic balance parallel to the front between coastal cold upwelled waters and the offshore warmer waters [14]. The front typically moves offshore to the shelf edge vicinity with prevailing upwelling-favorable winds [15, 16].

Generally, coastal upwelling cycles are asymmetric [17]. When upwelling-favorable winds relax, coastal temperatures become warmer, not only by a return of the water previously displaced offshore, but also as a result of poleward advection in a coastal current. Poleward flows of warm waters have been reported on the western Iberian inner shelf during relaxations in spring [3] and summer [18, 19]. These transient poleward shelf flows are distinct from the offshore IPC and have a variety of causes. In the summer, coastal poleward flows may be forced by alongshore density gradients, alongshore differences in wind forcing and by interaction of the flow with topographic features [9, 20–22].

During periods of high continental runoff, the resulting buoyant plumes contribute to the coastal poleward flow [23]. Under downwelling conditions, the plume waters from the rivers to the south may reach and enter the Rías Baixas, as surface fresh waters accumulate inside and warm through shortwave radiation [24]. In subsequent upwelling events, these waters are exported to the shelf, with lower salinity and higher temperature than the locally upwelled waters [3, 25].

This paper presents the main results from an oceanographic survey NW of the Iberian Peninsula between Cape Silleiro and Porto, covering the inception of upwelling in July 2009. We examine the shelf circulation in response to the atmospheric forcing over a sequence of upwelling and relaxation cycles. The presence of the Iberian Poleward current in the slope and shelf during the upwelling season is also discussed.

## Data and methods

**Fig 2:**
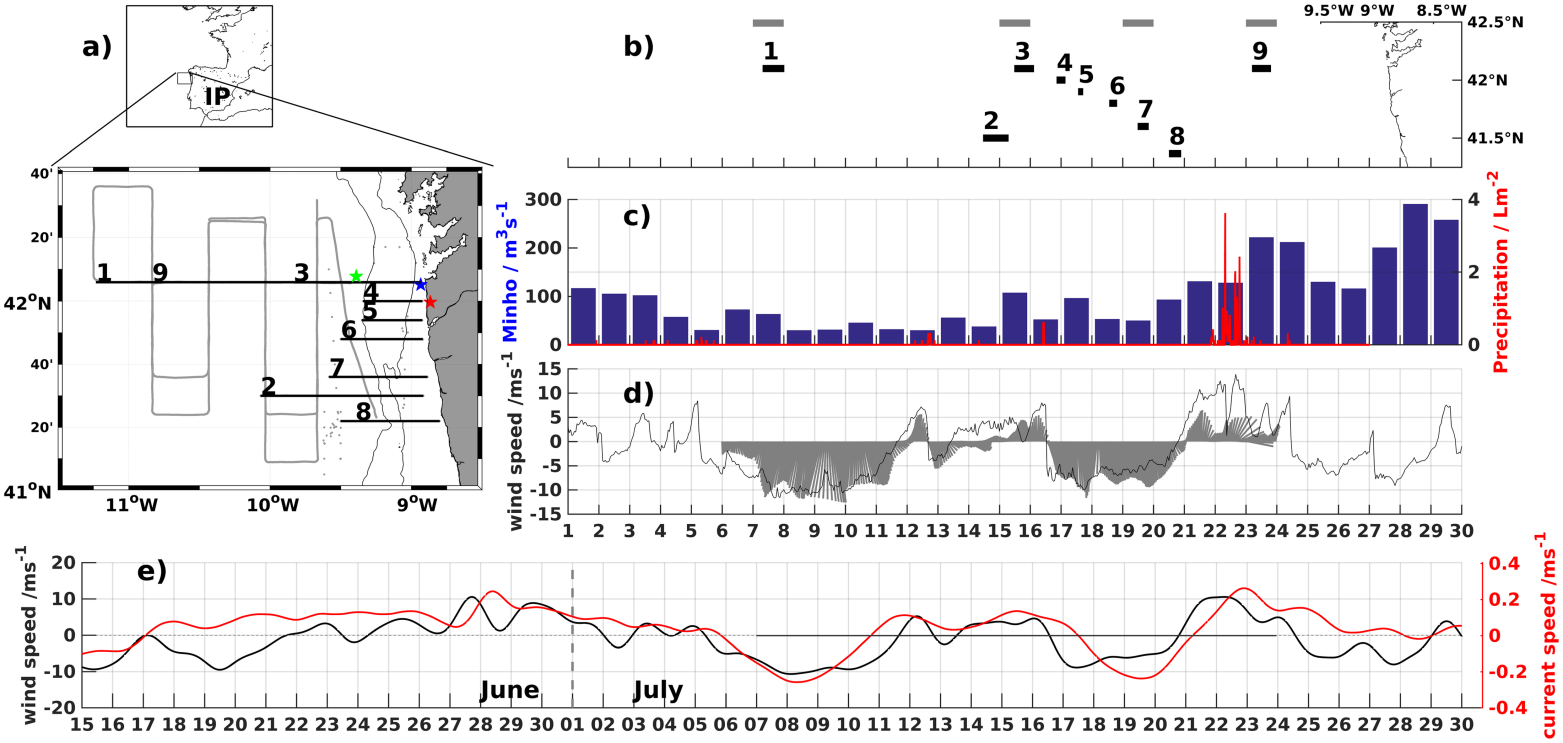
Location and time of observations. a) Study area, with gray line representing the SeaSoar path and gray dots the CTD stations. Zonal CTD transect (black lines) numbers 1 to 9 are indicated at their western end. Stars represent the position of *Puertos del Estado* Buoy (green), moored ADCP (blue) and Castro Vicaludo meteorological station (red). IP labels the Iberian Peninsula. The 100-m and 200-m bathymetric are shown in dark thin lines. b) Time-series with latitude of the numbered transects (black lines) and indication of the day of the satellite image (gray lines). c) precipitation at Castro Vicaludo Meteorological station and river Minho outflow, d) Hourly meridional winds from WRF simulation at the moored ADCP location and stick plot of filtered winds registered on the research vessel meteorologic station. e) 33-hour filtered meridional winds from WRF simulation and barotropic meridional currents from the moored ADCP.

From 7 to 23 July 2009, a cruise took place off the NW Iberian margin early in the upwelling season, on board the Research Vessel (RV) *Sarmiento de Gamboa* (Fig 2). Two hydrographic surveys were performed offshore of the 500 m isobath (gray line in Fig 2a) with continuous tows of a *Seabird 911+* conductivity-temperature-depth (CTD) probe on an undulating vehicle (SeaSoar) during the days 7-9 July and 21-23 July. The undulator was towed at 8 knots and cycled from near the surface down to 450 m depth providing a horizontal resolution of 3-4 km.

At the CTD stations, a General Oceanics rosette equipped with a *Seabird 911+* CTD and both upward and downward looking 300 kHz lowered acoustic doppler current profilers (LADCP), was lowered to a maximum of 600 m depth. Zonally oriented transects of salinity, temperature, plus alongshelf and across-shelf currents were obtained from the coast to the continental slope. In Fig 2b the transects analyzed in this study are ordered chronologically from 1 to 9.

**Fig 3:**
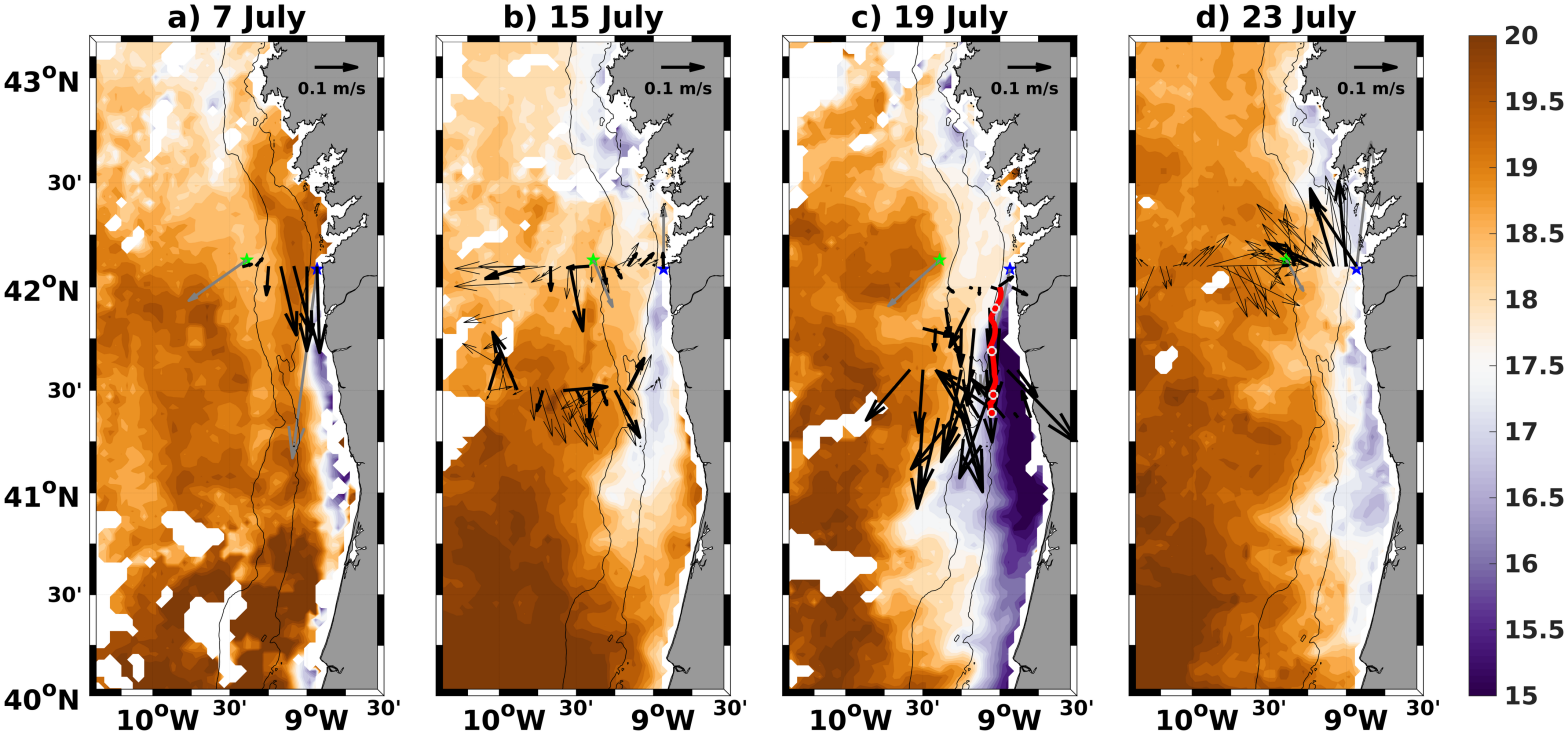
Maps of the SST from AVHRR satellite data on the days marked in Fig 2b, with instantaneous currents measured along the research vessel path from lADCP (bold arrows) and vmADCP (thin arrows), when available, between 50 m and 80 m depths. The 100 m and 200 m bathymetry are represented in black. Moored ADCP and the *Puertos del Estado* Buoy are located at the dark blue and green stars respectively. Mean currents for each day at 10 m depth from the moored ADCP and 3 m depth from the *Puertos del Estado* Buoy are represented by gray arrows. On panel c), the currents correspond to the period from 17 to 20 July, and a red line shows the trajectory of a surface drifting buoy (starting in the north), with white circles marking its location at 00h00 of each day (vmADCP data was omitted here for better visualization).

At 9:00 on 17 July a drifter buoy was launched around the position of the 100 m isobath at 42°N, and was recovered at 6:00 on 21 July (red line in Fig 3b). The buoy, equipped with GPS and Iridium communications, was drogued at approximately 10 m depth. The zonal transects number 4 to 8 were performed to follow its path in succession from north (42°N) to south (41.4°N), covering the continental shelf. This sampling methodology was designed to follow approximately the same water parcel during the upwelling event described below.

Transects number 1, 3 and 9 were made in front of Cape Silleiro, on 7 July, 15 July and 23 July respectively (Fig 2b). Transects 1 and 9 included 7 stations on the shelf and were continuous with the offshore zonal SeaSoar transect. Transect 1 extended to 11.25°W and transect 9 to 10.83°W. Transect 3 had 11 CTD stations between 9.45°W and the coast. Transect number 2 was made on day 14 and was located at 41.5°N, between 10.07°N and the coast.

A RDI 75 kHz Broadband Acoustic Doppler Current Profiler (vmADCP) mounted on the hull of the research vessel registered the current from 24 m to a maximum depth of 750 m from 15:00 of 8 July to the end of the cruise. The data were averaged in 8-m vertical bins below 24 m depth, and over 15 minutes intervals in time.

Underway data were logged on board the vessel from an Aanderaa meteorological station at 10 m height, a Seabird thermosalinometer sampling water pumped from 5 m depth and the GPS system for navigation. Noisy spikes were removed from all underway data with a 1-h window running mean.

The evolution of currents in the water column at the shelf mooring off Cape Silleiro (blue star in Fig 2a, -8.93°W; 42.08°N) was obtained in 3 m bins from an upward looking 300 KHz SonTek Acoustic Doppler Profiler moored at 72 m depth (hereafter named mADCP). After excluding outliers and anomalous values, depth-averaged alongshore current was calculated and low-pass filtered with half-power at 33h.

Currents measured at 3 m depth with a RDI/UCM-60DL on the Seawatch buoy off Cape Silleiro (42°7.8’N, 9°23.4’W; green star in Fig 3) were provided by Puertos del Estado (www.puertos.es).

Hourly wind speed and direction at 10 m above sea surface, from a 3-km resolution WRF configuration downscaled from the ECMWF reanalysis (ERA-interim) for the Iberian Peninsula, were provided by Meteorology and Climatology Group of the University of Aveiro (http://climetua.fis.ua.pt). The performance of WRF simulations were validated in the open ocean by [26] and by the comparison with the onboard meteorological system (Fig 2d).

Advanced Very High Resolution Radiometer (AVHRR) data of the NOAA were made available by the EUMETSAT Ocean & Sea Ice Satellite Application Facility (http://www.osi-saf.org). SST estimates were provided for 10:00 and 20:00 each day at a resolution of ∼2 km [M-F/CMS, 2009]. SST maps were retrieved for the days 7, 15, 19 and 23 July, marked with gray lines in Fig 2b. These four days are representative of the oceanographic conditions during the hydrographic transects with maximum possible image availability. The SST climatology of July was calculated using the same data set from years 2001 to 2016 (Fig 1).

Precipitation measured every 10 minutes at the Castro Vicaludo meteorogical station was provided by MeteoGalicia located at 8.86°W, 41.99°N (green star in Fig 3). Daily mean flow measured at the Frieiras Dam, representative of river Minho outflow, was provided by Confederación Hidrografica Miño-Sil (www.chminosil.es).

## Results

### General cruise conditions

Four distinct periods of wind forcing were identified during the cruise (Fig 2d). Between 5 and 11 July, an upwelling period was observed with northerly winds gradually strengthening to about 10 m/s on 8 July. On 11 July the wind weakened and shifted to a westerly direction, changing to a southerly direction on 12 July, at the start of a relaxation period with weak variable winds. This period lasted until mid 16 July, when the wind reverted from southerly to northerly, starting the second upwelling period. During this period, wind strength rapidly reached near 10 m/s, where it remained until late 19 July, when it started to weaken again. On 21 July, winds shifted to southerlies, peaking at almost 15 m/s on 22 July in the WRF record. On this day high precipitation rates were registered (accumulating 35 Lm^−2^ on 22 July) and the daily mean river Minho outflow was higher than 150 *m*^3^*s*^−1^(Fig 2c).

Fig 2e shows the low-pass filtered alongshore component of the sub-inertial currents and wind for the second half of June and July at the moored ADCP location. Weak and variable winds prevailed in the weeks preceding the study period, while the shelf flow was poleward with currents between 0.1 m/s and 0.2 m/s. During the first and second upwelling periods the current became equatorward within a few hours, in response to the northerly wind, with maximum alongshore current of about -0.2 m/s. During the southerly wind event starting 21 July, poleward flow velocities were recorded by the mADCP by 23 July. For the period depicted in Fig 2e, alongshore wind and current components were significantly correlated (R = 0.62,p = 0) with wind leading current by 12h.

A synoptic view of the circulation regime can be seen in a combination of satellite images and currents on the shelf, measured by the vmADCP and mADCP on Fig 3. The early stage of the first upwelling period (5 to 11 July) is shown in Fig 3a where a developing equatorward flow and the first stage of outcropping of cold waters associated with coastal upwelling is evident. After the upwelling period has passed, in Fig 3b, the alongshore surface front was located near the shelf edge. Resulting from the wind inversion to southerly on 12 July, a warm inner-shelf poleward flow was set up from 13 to 15 July between the cold recently upwelled waters and the shore, particularly at 41°N. On the inner shelf, currents were generally poleward while near the slope they were equatorward. The second upwelling period (16-21 July) is represented in Fig 3c, where the strong temperature front between offshore waters and recently upwelled onshore waters is seen. Measured currents were mostly southward across the sections. The Lagrangian drifter drogued at 10 m depth was also moving southward along the 100 m bathymetry. The drifter accelerated during 18 and 19 July and slowed down on 20 July, in agreement with the pattern of the mADCP currents (Fig 2e) and the lADCP at nearby stations. The period of 21 to 23 July (represented in Fig 3d) is characterized by an inversion of the previous upwelling favorable conditions, and the setting up of a generalized poleward flow along most of the shelf with currents of 0.2 m/s. In spite of the prevailing downwelling conditions a remnant of the previous upwelling pulse is still visible as a band of cold water at the coast.

### Water masses

**Fig 4:**
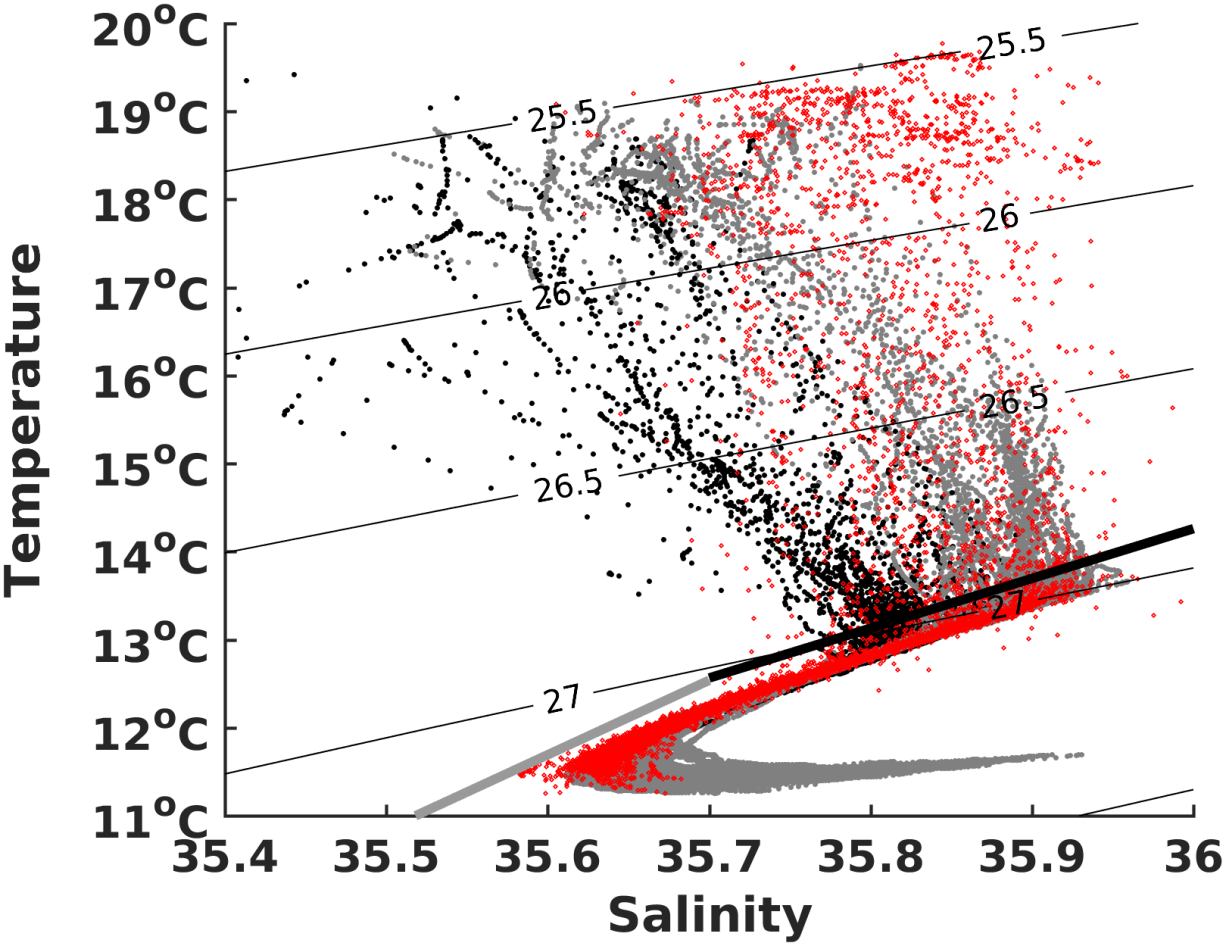
TS diagram of CAIBEX cruise hydrography observations. SeaSoar (red) and CTD measurements (black—on the shelf, gray—profiles deeper than 200-m depth).

Fig 4 shows the TS-diagram of all CTD casts and SeaSoar profiles. Surface waters, with densities between 25.5 and 27 kg/m^3^, increased in temperature and salinity from the shelf to offshore. Most profiles converge to the same water mass below 27 kg/m^3^, reaching the depth of the ENACWst (Eastern North Atlantic Central Waters—Subtropical). At this level, salinity maximums are observed, reaching 35.82±0.2 on the shelf and 35.9±0.5 offshore. The ENACWst density remains fairly constant for salinities higher than 35.7. Between this point and 35.6, all profiles contain a salinity minimum as the signature of the subpolar origin waters (ENACWsp). In CTD profiles on the slope, a rise of salinity and temperature with depth in the lower part of the ENACWsp below 500 m depth, reveals the influence of the underlying Mediterranean waters.

### The IPC

**Fig 5:**
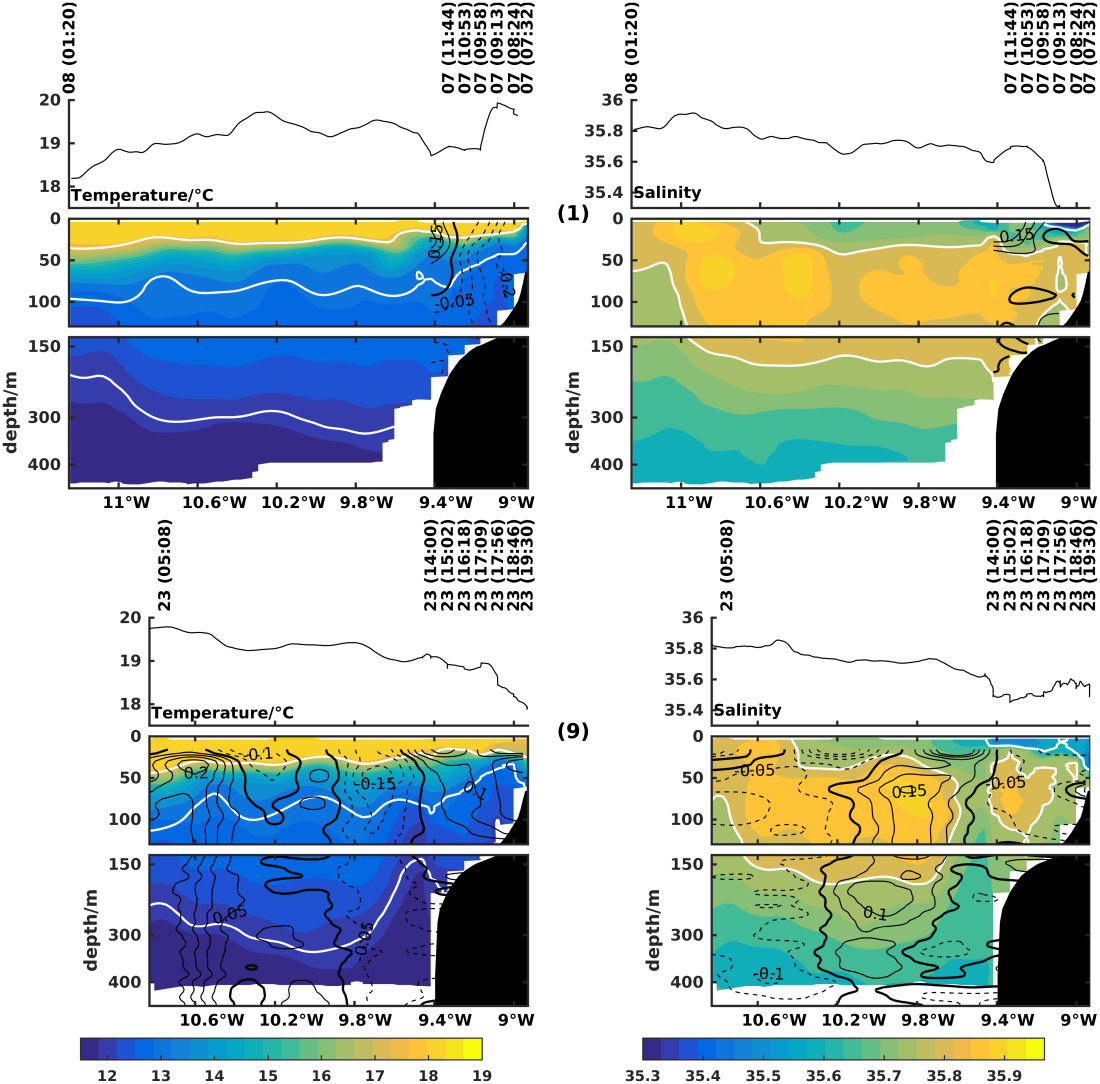
Zonal transects numbered 1 and 9 of temperature (left column) and salinity (right column) off Cape Silleiro (42,10°N). Day and time of each CTD cast and the underway surface temperature and salinity from the thermosalinograph are shown at the top. Dark contour lines represent along-shore (left column) and cross-shore (right column) currents in m/s measured with lADCP or vmADCP, when available. White contour lines are the 26 kg/m^3^ and 27 kg/m^3^ isopycnals on the left plots and the 35.6 and 35.8 isohalines on the right.

The poleward flow off the Western Iberian Margin is associated with a subsurface salinity and temperature maximum and a downward slope towards the coast of isotherms and isohalines. The characteristics of the top 400 m of the IPC (Fig 5) were evaluated at the beginning and end of the cruise, on 7 and 23 July respectively, with the two long hydrographic transects off Cape Silleiro (transects 1 and 9 in Fig 2).

In transect 1, 7 July, below ∼70 m depth both the thermal and haline fields tilt downward towards the coast, in association with subsurface poleward flow across this section between 11°W and the slope. Above 70 m depth the opposite behavior is observed for the isopycnal field, with a tendency to tilt upward in this layer. At ∼10.2°W a mesoscale anticyclonic feature is visible.

Near the end of the cruise, 23 July, a shorter transect made at the same latitude, showed similar patterns to the initial transect from offshore 10.6°W to 9.8°W near the slope. The observed meridional flow was mainly poleward from the western extreme at ∼10.8°W to 9.8°W, associated with the downward slope of the isopycnals. This poleward flow was centered at ∼10.7°W with a maximum velocity of 0.2 m/s. From 9.8°W to 9.4°W the slope of thermal and haline fields changed and upwelling was observed below 50 m depth. Accordingly equatorward flow was observed near the upper slope, with maximum velocity around 0.15 m/s. The dynamics associated with this flow are discussed below.

### Hydrography and currents of the distinct periods

Four distinct periods were identified, as upwelling-favorable periods were interleaved with relaxation and downwelling. The first upwelling period of the cruise, 5 to 11 July was sampled only once on the shelf in transect 1. A relaxation period between 11 and 16 July was sampled by transects 2 and 3. The second upwelling period, 16 to 20 July, was sampled in 5 different latitudes by across-shelf transects 4 to 8. From 21 to 24 July, a downwelling period, the shelf was sampled once, on transect 9.

#### 5-11 July: First upwelling period

The first upwelling favorable period followed a two week period of poleward flow and weak winds (Fig 2). Cold waters surfaced for the first time in AVHRR satellite images nearshore on 7 July, and gradually extended to the shelf edge until 10 July (not shown). Northerly winds were strongest in this period of offshore advection of the front, and relaxed rapidly on 11 July.

**Fig 6:**
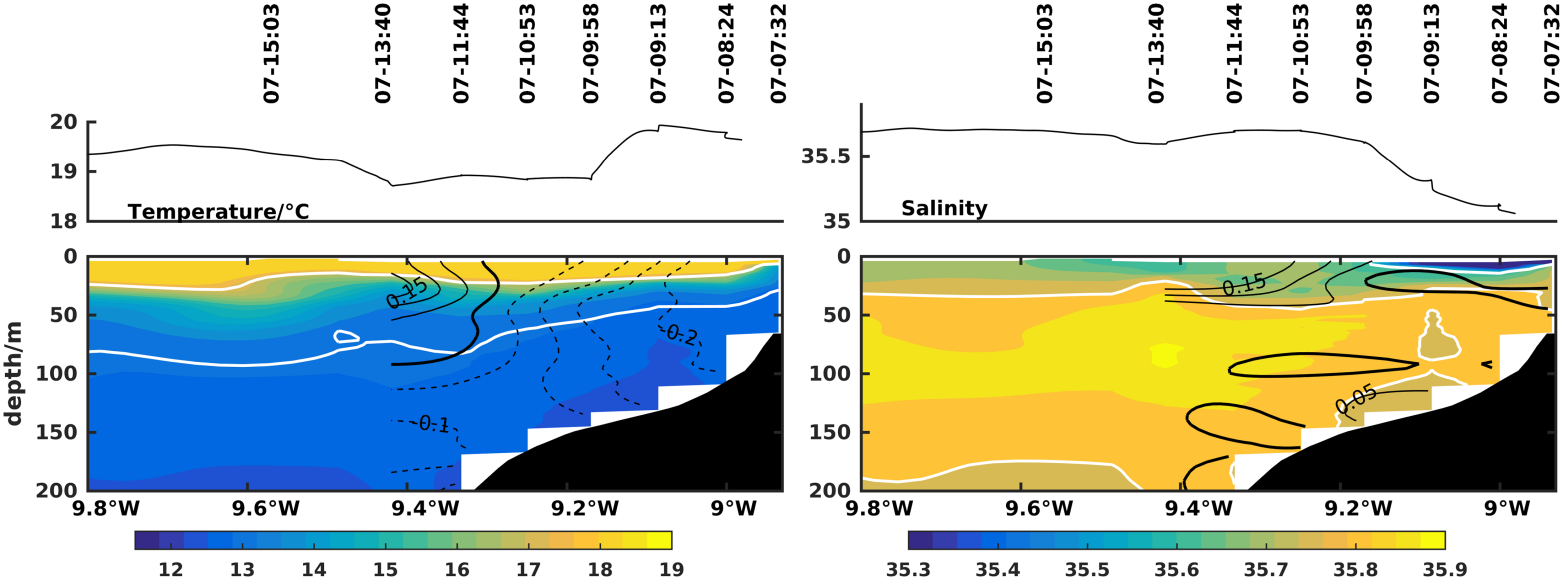
Zonal transect 1 of temperature (left column) and salinity (right column) off Cape Silleiro (42.1°N). Day and time of each CTD cast and the underway surface temperature and salinity from the thermosalinograph are on the top. Contour lines represent along-shore (left column) and cross-shore (right column) currents in m/s measured with lADCP or vmADCP, when available. White contour lines are the 26 kg/m^3^ and 27 kg/m^3^ isopycnals on the left plots and the 35.6 and 35.8 isohalines on the right.

Fig 6 shows the top 200 m of transect 1 inshore of 9.80°W sampled on 7 July. The 27 kg/m^3^ isopycnal rose from about 80 m depth offshore to less than 50 m nearshore. The thermocline was also shallower than 10 m nearshore, although not outcropping the surface. A fresher (<35.5) and warmer water (∼19.8°C) plume was observed at the surface of the inner shelf. The surface plume extended to 9.1°W as a warm water patch in the SST AVHRR image (Fig 3a) flowing out from Ria de Vigo to the shelf.

During the upwelling event, the currents on the shelf flowed equatorward, most strongly on the inner shelf (>0.2 m/s; Fig 6). Nevertheless on the outer shelf, the top 100 m flowed poleward with a maximum of 0.15 m/s. The zonal component was near-zero everywhere.

#### 11-16 July: First relaxation period

**Fig 7:**
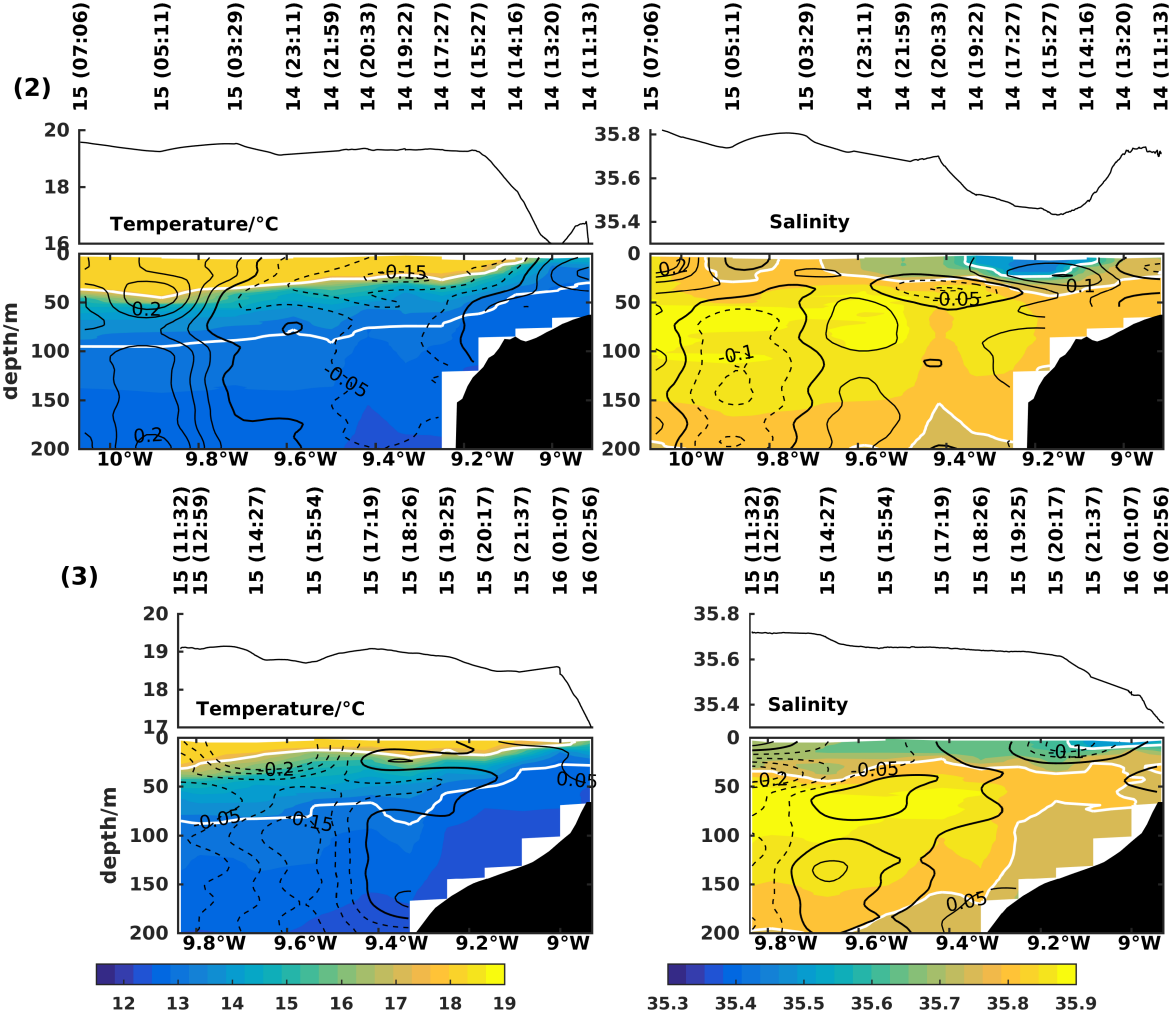
Zonal transects 2 and 3 of temperature (left column) and salinity (right column) off Cape Silleiro (42.1°N). Day and time of each CTD cast and the underway surface temperature and salinity from the thermosalinograph are shown at the top. Contour lines represent along-shore (left column) and cross-shore (right column) currents in m/s measured with lADCP or vmADCP, when available. White contour lines are the 26 kg/m^3^, 27 kg/m^3^ and 27.1 kg/m^3^ isopycnals on the left plots and the 35.6 and 35.8 isohalines on the right.

Resulting from the wind relaxation of 11 to 16 July, set up of poleward flow was registered by the mADCP from 11 July onward (Fig 2e) with typical values of 0.1 m/s. On 14 July this flow was evident in transect 2 (Fig 7a) at latitude 41.5°N, coexisting with an upwelling front at longitude 9.1°W, an equatorward flow over the upper slope and the IPC offshore of 9.7°W. Associated with the expected shoreward retreat of the upwelling surface front and with the onset of weak southerly winds, a shoreward surface flow is visible centered at 9.2°W, coincident with the plume of fresh waters. One day later, at 42.1°N latitude in transect 3 (Fig 7b), weak poleward flow of less than 0.1 m/s was still observed over the shelf while offshore of the shelf break, equatorward flow was prevalent.

#### 16-20 July: Second upwelling period

**Fig 8:**
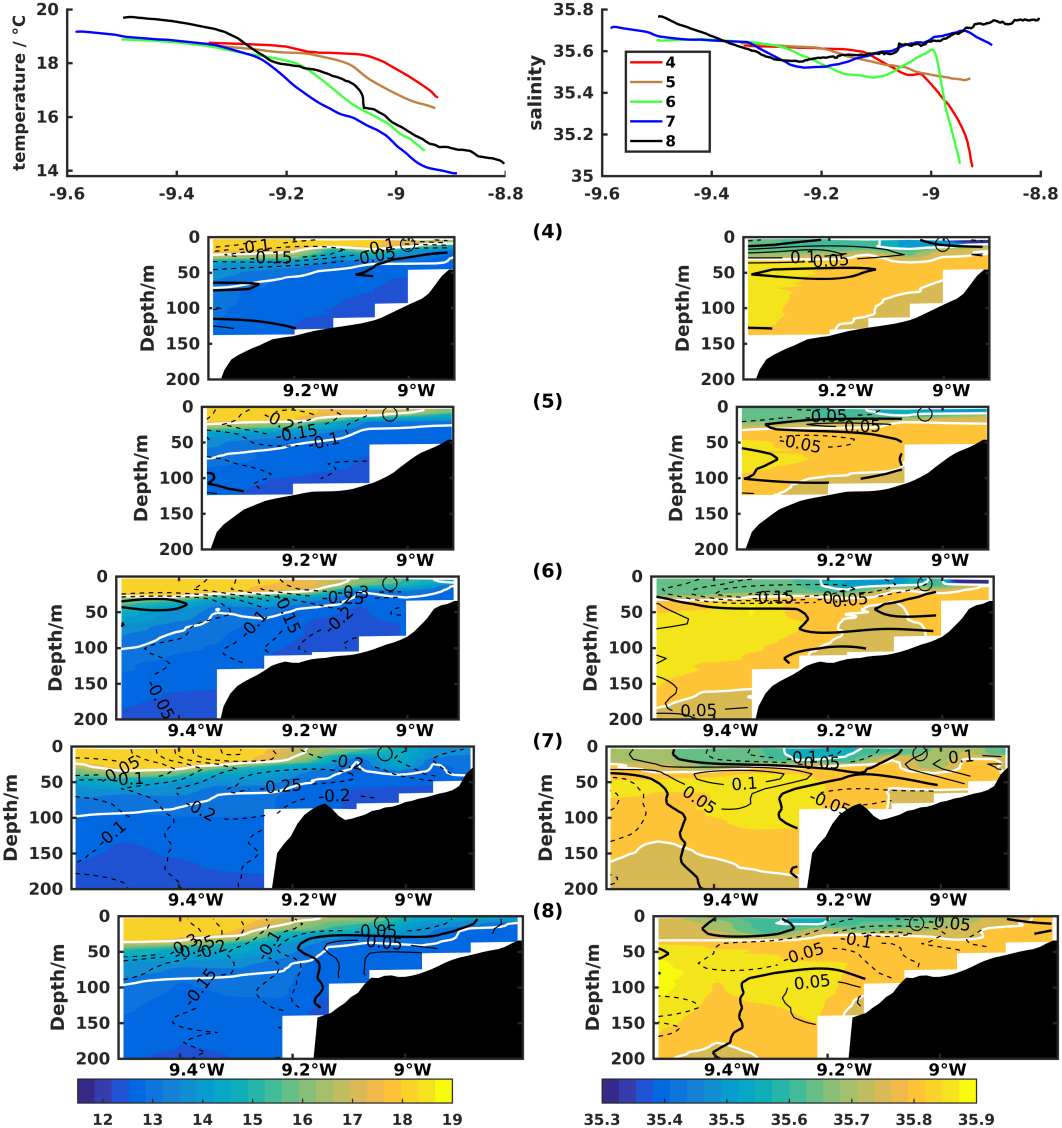
Sequence of zonal transects 4 to 8 of temperature (left column) and salinity (right column). The underway surface temperature and salinity from the thermosalinograph are shown at the top. Contour lines represent along-shore (left column) and cross-shore (right column) currents in m/s measured with lADCP or vmADCP, when available. White contour lines are the 26 kg/m^3^ and 27 kg/m^3^ isopycnals on the left plots and the 35.6 and 35.8 isohalines on the right. At each transect, a circle located at 10 m depth represents the position of the drifter buoy.

During this upwelling period, a drifter drogued at 10 m depth was released on 17 July in mid-shelf at 42°N. Following the path of the drifter buoy, a sequence of 5 across-shelf transects (numbers 4 to 8) was conducted (Fig 8). Since the drifter buoy had a quasi-Lagrangian nature, the transects sampled approximately the same water parcel at 10 m at the longitude where they crossed the buoy path. The drifter followed the 100 m depth contour in the equatorward direction but was also affected by tidal and inertial oscillations. On the last day, 20 July, the drifter slowed down as the northerly winds relaxed.

The northernmost transect at latitude 42°N (4, of Fig 8) was sampled around 15h after the beginning of the upwelling pulse. At this time an equatorward flow was present at the surface, while poleward flow was still observed below ∼40 m depth. As a result of the preceding relaxation period, a stratified surface layer was observed across the shelf, with evidence of an incipient upwelling near the coast. SST measured by the thermosalinograph decreased 1°C near the coast. Beneath the surface, the temperature and salinity fields were tilted upward, and central waters were evident on the shelf bottom (<13°C temperature, <35.6 salinity). The surface salinity field was strongly influenced by a river plume. While offshore flow associated with the Ekman layer appeared at the surface, a compensating onshore flow was observed to transport salty waters onto the shelf below.

Still on 17 July, transect 5 at latitude 41.9°N shows the development of the surface equatorward flow with maximum velocities of 0.2 m/s. The development of upwelling is noticeable in the thermal field, with the cooling of the inner shelf surface waters and the establishment of a surface front. The cross-shore field exhibited a surface offshore flow.

The typical picture of fully developed upwelling was first seen in transect 6 on 18 July at 41.8°N latitude. At the surface, equatorward flow was observed, reaching maximum values of 0.3 m/s above a weakly sheared equatorward flow with values over 0.2 m/s. By this day the 27 kg/m^3^ isopycnal had broached the surface, and the 26 kg/m^3^ isopycnal outcropped mid-shelf as part of a well developed front being advected offshore at 0.2 m/s by a 40 m thick surface flow. At depth, a weak onshore return current was observed, as expected in a well developed upwelling phase. The surface plume of low salinity, previously adjacent to the coast, now extended to 9.2°W and was broken into two at around 9.05°W though stronger on the inner shelf. At deeper levels, the onshore flow advected salty waters (35.8-35.9) onto the continental shelf.

By 19 July, at 41.6°N latitude (transect 7), continued upwelling had advected the surface front offshore to the shelf break. The band of equatorward flow also extended offshore to the shelf break, while coastal alongshore currents decreased from -0.3 m/s in transect 6 to -0.1 m/s here. The offshore flow in the Ekman layer decreased while a complex cross-shore distribution developed below. The fresh water surface tongue extended further offshore with the salinity minimum observed at about 9.2°W just offshore of the shelf break.

At the end of the upwelling pulse, transect 8 was sampled at 41.36°N latitude, late 20 July. The surface seaward advection of the upwelling front seen in the previous two transects ceased simultaneous with the start of the northerly wind relaxation. Poleward flow was rapidly established on the inner shelf and below 30 m on the middle and outer shelf. Offshore of the shelf break the equatorward flow persisted in the surface layer. Resulting from the wind relaxation, the saltier waters at deeper levels over the shelf were advected weakly seaward.

#### 21-24 July: Second relaxation period

**Fig 9:**
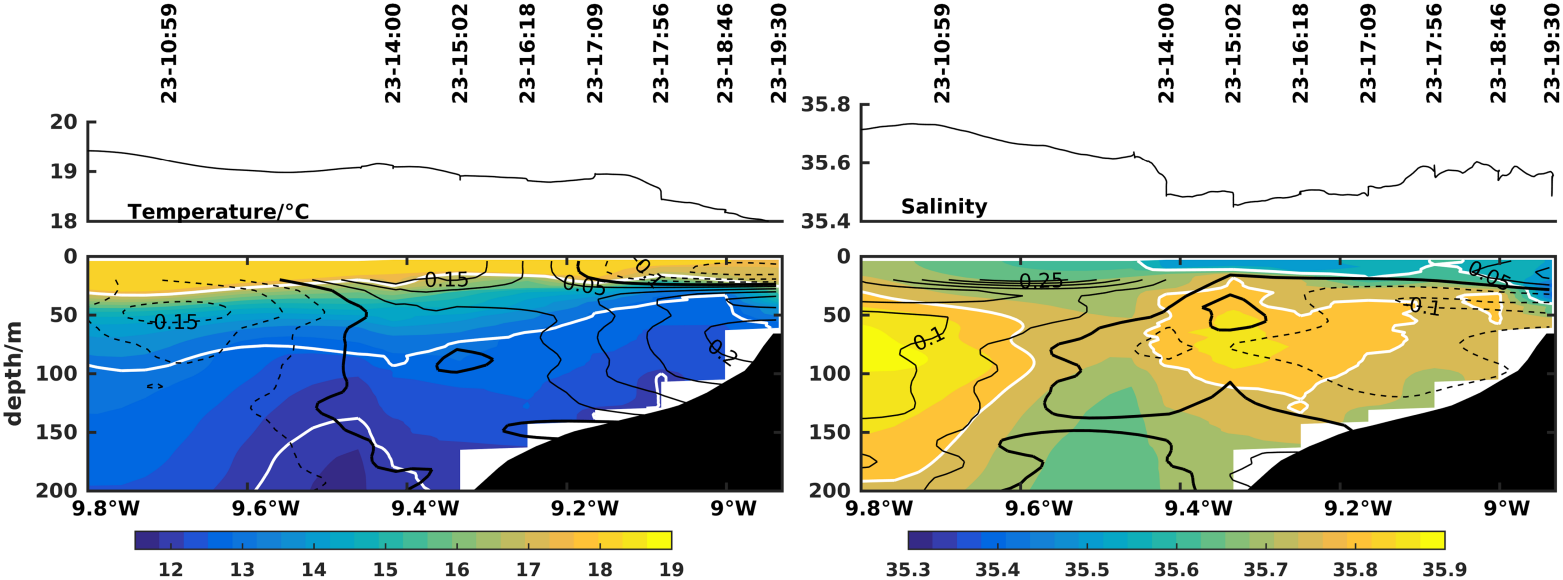
Zonal transect 9 of temperature (left column) and salinity (right column) off Cape Silleiro (42.1°N). Day and time of each CTD cast and the underway surface temperature and salinity from the thermosalinograph are shown at the top. Contour lines represent along-shore (left column) and cross-shore (right column) currents measured with lADCP or vmADCP in m/s, when available. White contour lines are the 26 kg/m^3^, 27 kg/m^3^ and 27.1 kg/m^3^ isopycnals on the left plots and the 35.6 and 35.8 isohalines on the right.

During this period, intense southerly forcing produced in transect 9 at 42.1°N (Fig 9) set up of poleward flow across most of the shelf, as usual coexisting with equatorward flow offshore of the shelf break. In the cross-shore flow, Ekman coastal convergence induced local downwelling of a low salinity plume down to ∼50 m depth, while weak offshore return flow was observed below this layer. A closed salinity maximum of 35.8 separate from the offshore IPC was observed at the outer shelf.

## Discussion

In the present study a situation of repeated upwelling spin up and relaxation cycles was observed, consisting in a sequence of development, offshore advance and retreat of upwelling fronts during nearly three weeks of July 2009. This situation corresponds to the first significant upwelling after the establishment of the surface layer stratification associated with the spring transition of that year. During the preceding two weeks, weak southerly winds prevailed. The oceanographic conditions on the shelf were characterized by poleward flow, sampled with an ADCP moored at -8.93°W, 42.08°N, at the 72 m isobath, with mean values of 0.1 m/s.

The first transect of the cruise took place on 7 July, two days into the first upwelling favorable (northerly) wind period. Within this short period, a nearly barotropic equatorward jet associated with upwelling was set up, with maximum values of 0.2 m/s at the mADCP position, although the signature of upwelling could not be seen at the surface, as no clear outcropping of isopycnals was observed.

From 11 to 16 July was the first period of relaxation with weak and mostly southerly winds. In spite of the lack of upwelling-favorable winds, the hydrographic fields still resembled those of the previous period with evidence of upwelled waters on the shelf. However, the reversal of the flow was observed on the shelf with prevalence of a weak poleward flow (0.05-0.1 m/s).

Another upwelling favorable period occurred between 16 and 20 July. The observations show the quasi-Lagrangian sequence of the upwelling cycle south of Cape Silleiro, following a drifting buoy through the event. The shift from weak shelf currents to a fully developed equatorward flow was observed early in this upwelling cycle. On 20 July, wind began to relax and, in the southern transect, a poleward flow was observed nearshore extending to the mid-shelf bottom while equatorward flow was located offshore of the shelf.

From 21 to 23 July the second relaxation period occurred with strong downwelling favorable (southerly) winds. On 23 July, poleward flow was predominant on the shelf and coastal convergence was noticeable with a low salinity plume constrained against the coast.

In July 2009, on the Western Iberian Margin north of 41°N, two upwelling events were able to force equatorward shelf flow when they were strongest. The interruption of the upwelling regime, either by downwelling-favorable winds or relaxation, allowed the almost immediate return to a shelf poleward flow. These circulation cycles characterize the upwelling season of the NWIM, as the upwelling-favorable winds occur as intermittent pulses of several days and do not persist long enough to impose a completely equatorward regime.

On the three occasions the research vessel sampled offshore of the slope (transects 1, 2 and 9), poleward flow was observed, either directly through vmADCP measurements or estimated through the density fields. These flows at the offshore region are associated with the Iberian Poleward Current, observed predominantly during Autumn/Winter [27–30], but also during the Spring-Summer transition [3]. The poleward flow during summer was found also in numerical modeling studies of the WIM [5]. The numerical study of [31], using realistic forcing, reports an IPC (for spring) with similar structure to our observations, i.e. spread further from the slope.

Around the upper slope region, equatorward flow, accompanied by a doming of cold and fresh water between 9.4°W and 9.6°W is observed from the second transect onward. This flow could originate at Cape Finisterre and then be advected in the equatorward direction along the upper slope [3]. Similar behavior was described by [18] based on satellite imagery to interpret the tongue of cold water extending from Cape Finisterre to Porto as a remnant of the equatorward jet of previous upwelling periods. Note that other authors report similar behavior for other locations of the WIM, namely [32] for Cape Roca, and [33] for Cape São Vicente.

The equatorward flow over the upper slope is characterized by a weak vertical shear intensified at the surface Ekman layer. It is likely that this equatorward flow influences the location of the IPC, keeping it offshore in the early upwelling season as suggested by the numerical study of [5], while later in the upwelling season the IPC usually develops under the upwelling jet in the upper slope [31].

The upper slope equatorward flow separates two subsurface high salinity cores, one offshore associated with the IPC and another over the mid and outer shelf. This is particularly visible at the end of the cruise at longitude ∼9.5°W (transect 9). Furthermore, the dome of temperature and salinity fields associated with the upper slope equatorward flow is located offshore of the upwelling front. On the other hand, its SST signature is not clearly discernible by satellite imagery, as it is in [18] examples.

On the shelf the circulation is mainly wind driven though pressure gradients and buoyancy input from river outflows play a role. During the two periods of northerly winds, an upwelling front developed along with its corresponding equatorward jet and across-shelf circulation. The lagrangian drifter released in the frontal region early in the upwelling period of 16 to 20 July proceeded to move equatorward following the 100 m isobath, advected under the influence of the upwelling jet.

The surveyed region, between Cape Silleiro and Porto, was a center of upwelling in July 2009, as is often the case [8] evidenced by the July mean SST (Fig 1). The existence of this upwelling center could be associated with two factors: i) coastal orientation nearly parallel to the prevailing upwelling-favorable winds (NNW) and ii) the variable alongshore shelf bathymetry. In the region of the upwelling center, the shelf widens in the equatorward direction. [10], using a barotropic, linear and steady potential vorticity equation, showed that the onshore transport of deep upwelled waters, primarily through the bottom layer, enhances where the shelf widens in the direction of the upwelling jet, hence upwelling is intensified in these regions. The generation of upwelling centers was discussed in the baroclinic case by [34] for the northeastern South China Sea, reinforcing the association of upwelling centers to regions of widening shelfs.

This upwelling center may induce the establishment of a poleward inner shelf flow once the northerly winds relax, as is the case of the Northern Californian shelf from Point Reyes to Point Arena [9]. These authors show that in the presence of an upwelling center, a poleward gradient in density sets an equatorward pressure gradient that induces poleward flows, maintained when upwelling favorable winds relax. This mechanism may explain the trend to poleward flow during the relaxation period of 12 to 16 July. This mechanism could also explain the coastal poleward flow described by [18] associated with wind relaxation at the end of the upwelling season.

On the other hand, during the second relaxation period, the observed poleward flow in the shelf seems to be associated with downwelling-favorable winds, in accordance with the classic downwelling theory. Other example of wind driven downwelling flow in the region is provided by [3]. In this relaxation period, the coastal poleward flow advected a low salinity plume and downwelling promoted its convergence and coastal trapping near Cape Silleiro. This structure is unusual in the summer although it was already documented in similar systems such as the Columbia River plume [35] and supports regional modeling experiments such as [36]. In the early stages of upwelling, exporting of plumes from Rias Baixas, can briefly hinder the surfacing of upwelled waters, such as in Fig 6. During the upwelling periods, the buoyant plume is advected southward and offshore across the shelf, as was the case in [37] further south on the WIM and was observed in the sequence of transects in Fig 8. The shoaling of the exported offshore plume results in the confinement of plankton in these nutrient rich waters [38].

The response of the circulation on the shelf to transient events of upwelling, relaxation and downwelling is linked to the ecosystem dynamics. In terms of advection patterns, upwelling pulses and downwelling events have been shown important for the bi-directional transport of species of phytoplankton between the Rias Baixas and central WIM [39], responsible for the alongshore spreading of harmful algal blooms.

As [37] reported during an upwelling event, the upwelling pulses regularly bring cold, fresh and nutrient-enriched waters to the surface, driving the biological cycles. In general the upwelling front separates the oligotrophic offshore ecosystem characterized by low biomass of small phytoplankton species (mainly cyanobacteria) located at the bottom of the surface mixed layer in a subsurface chlorophyl maximum (20-50m depth), from the inner coastal waters, where the species distribution are mostly large microplakton (diatoms and dinoflagellates) especially during the upwelling relaxation [40–42].

The physical processes dicussed above are also linked to the nutrient and oxygen cycles. In the presence of upwelling, the surface nutrients are depleted by the phytoplankton, then replenishment at depth by microbial remineralization of the sinking organic matter [43], which consumes the oxygen at deeper levels. Minimum oxygen concentrations were observed in the bottom layers of the shelf in this cruise (not shown) in line with the [37] results. In the offshore olygotrophic waters, sub-surface maximums of oxygen are associated with photosynthesis of phytoplankton.

## Conclusion

An 18 days long oceanografic survey took place in the NW Iberian Margin in July 2009, sampling the shelf and offshore, allowing a synoptic view of several events, including a novel quasi-Lagrangian sampling of an upwelling event. The cruise observations were complemented by an upward looking ADCP moored near Cape Silleiro at 72 m depth.

**Fig 10:**
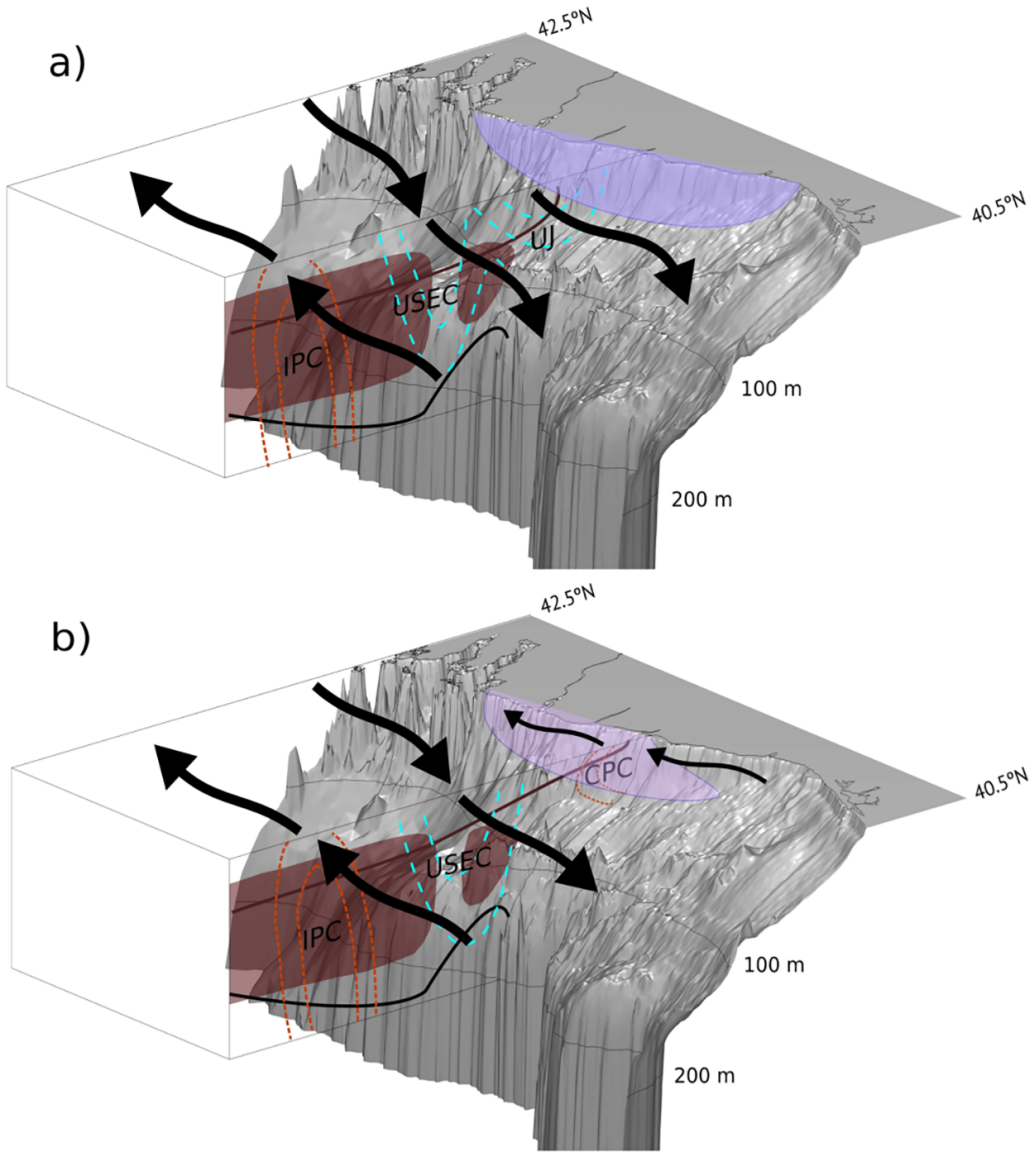
Conceptual schemes of circulation under (a) upwelling regime and (b) relaxation regime. The prevalent currents are depicted on the transect by the dashed countours in red (poleward flowing) and blue (equatorward flowing) and identified with the acronyms: Iberian Poleward Current (IPC), Upper Slope Equatorward Current (USEC), Upwelling Jet (UJ) and Coastal Poleward Current (CPC). On the transect, dark lines represent the preceived field of isothermals and reddish areas mark location of central waters with subtropical origin. At the surface, the perceived paths of currents are marked as arrows, and the upwelling center as the blue (a) and purple (b) shading, representing a stronger and weaker temperature anomaly, respectively.

The results of this cruise show the rapid response of the shelf circulation to changes in alongshore wind both in developing an equatorward flow regime (following the classical theory of upwelling), and the set-up of poleward flow associated with wind relaxation. These two circulation regimes are schematically represented in Fig 10. Excepting the shelf, the circulation was similar to both regimes, with the IPC located offshore of an upper-slope equatorward current, both associated with the subsurface isothermal slope.

The scheme of Fig 10a corresponds to a regime typical of prevailing northerly winds, including a center of intense upwelling (with an associated temperature front) and an upwelling jet observed throughout the shelf, circulating alongside the upper-slope equatorward current. Another regime is associated with the relaxation of upwelling favorable winds (Fig 10b), which is characterized by a poleward flow along the shelf. Warm waters are carried nearshore intruding into the previously generated cold upwelling center. The system of currents reported here, at the timescale of the shelf circulation response to wind forcing, confirms previous findings in the literature. Their further investigation in this region is relevant from the point of view of marine ecosystem management, as these transport processes strongly influence the biogeochemical properties and the alongshore distribution of harmful algal blooms, larvae and pollutants along the coast of the NW Iberian Margin.

## Supporting information

S1 Dataset Complementary data used to reach the conclusions drawn in the manuscript. Includes the relevant observations from the CAIBEX project and the wind data from the WRF simulation.
(ZIP)
