# Correction: Transient response of the Northwestern Iberian upwelling regime

**DOI:** 10.1371/journal.pone.0199806

**Published:** 2018-06-21

**Authors:** Nuno Gonçalo Ferreira Cordeiro, Jesus Dubert, Rita Nolasco, Eric Desmond Barton

In the Funding section, the reference number for the FEDER funding is missing. The reference number is: POCI-01-0145-FEDER-007638.
